# Comment #2 on “Differences in Ventilatory Threshold for Exercise Prescription in Outpatient Diabetic and Sarcopenic Obese Subjects”

**DOI:** 10.1155/2018/4026463

**Published:** 2018-11-28

**Authors:** Goran Kuvačić, Luca Paolo Ardigò, Johnny Padulo

**Affiliations:** ^1^Faculty of Kinesiology, University of Split, Split, Croatia; ^2^School of Exercise and Sport Science, Department of Neurosciences, Biomedicine and Movement Sciences, University of Verona, Verona, Italy; ^3^University eCampus, Novedrate, Italy; ^4^Research Laboratory “Sport Performance Optimization”, National Center of Medicine and Sciences in Sport (CNMSS), Tunis, Tunisia

This journal published an original article entitled “Differences in Ventilatory Threshold for Exercise Prescription in Outpatient Diabetic and Sarcopenic Obese Subjects” [1]. Following a first letter regarding that article [2] and a response by its authors [3], some points still remain unclear. The aim of this second letter is to point out the unaddressed points and stimulate some thoughts.

We were waiting for an exhaustive response to our letter [2]. Yet, their response did not satisfy us completely. Therefore, we point out again only some issues previously raised but still not properly addressed [1, 3].

In *2.3. Maximal Effort and Individual Ventilatory Threshold*, the authors support the use of the chosen treadmill incremental protocol by means of a self-citation, which might be proper if only the provided self-citation would really describe the protocol. Yet, this is not the case, because the self-citation refers only to an undescribed *modified Balke protocol*. Therefore, the authors should describe the chosen treadmill incremental protocol and provide the original reference of the Balke protocol (the one used in the self-citation, “[27] B. Balke and R. W. Ware, “An experimental study of physical fitness of Air Force personnel” United States Armed Forces Medical Journal, vol. 10, no. 6, pp. 675–688, 1959,” would fit well). Furthermore, the authors did not provide any information on the correct treadmill use for scientific research (e.g., on speed calibration or slope setting [4]).

In *2.3. Maximal Effort and Individual Ventilatory Threshold*, we refer to the fact that the maximal effort and individual ventilatory threshold were assessed by a maximal graded exercise test on a treadmill according to individual abilities as previously done in another authors' study [5].

1. Supported by a bulk of literature [6], we absolutely disagree with the use of the Balke protocol (or any modified Balke protocol) for outpatient diabetic and sarcopenic obese subjects. And especially for pathological subjects, it is particularly important to achieve true V'O_2peak_ (which was the specific study's condition) [7]. Traditionally, in such subjects, V'O_2peak_ is achieved by administrating traditional graded protocols, but the suggested protocol in pathological subjects is—in by far most instances—the Naughton one [8], not the Balke protocol, even if there is a growing body of literature supporting the use of individualized (continuous) ramp protocols in pathological subjects [6], i.e., protocols completely different from the traditional graded ones, like the Balke protocol
2. There is no information about the chosen methodology test-retest precision of the measure [9]
3. About the treadmill use, the authors did not address our request to provide the device's brand and model (as commonly done in scientific literature). Neither did the authors provide any information about speed calibration or slope setting [4, 10]

In conclusion, we think that the attention of sport scientists should focus more on methodological issues. Sport scientists should aim as much as possible at both accuracy/precision of the measure and reduced exposure to errors. Acceptable accuracy and precision are essential requirements to perform sound research. Therefore, sport scientists should carefully choose among the available methods the most effective ones to pursue high accuracy/precision standards.
